# Routine Indwelling Urinary Catheterization Is Not Necessary During Total Hip Arthroplasty Performed Under Spinal Anesthesia

**DOI:** 10.1016/j.artd.2022.04.015

**Published:** 2022-05-28

**Authors:** Kurtis D. Carlock, Zachary D. Mills, Kyle W. Geiger, Paul A. Manner, Navin D. Fernando

**Affiliations:** Department of Orthopaedic Surgery and Sports Medicine, University of Washington, Seattle, WA, USA

**Keywords:** Urinary retention, Urinary catheterization, Foley catheterization, Total hip arthroplasty, Spinal anesthesia

## Abstract

**Background:**

Perioperative indwelling urinary catheterization remains common in patients undergoing total hip arthroplasty. This study sought to examine the effect of routine catheterization following total hip arthroplasty performed under spinal anesthesia on urinary complications.

**Methods:**

A total of 991 consecutive patients who underwent primary total hip arthroplasty under spinal anesthesia over a 4-year period were retrospectively reviewed. Major postoperative urinary retention (POUR) was defined as persistent retention following 2 straight catheterizations, which required postoperative indwelling catheter placement. Minor POUR was defined as retention that resolved following 1 or 2 straight catheterizations. Statistical analyses were used to compare outcomes between those who received a routine indwelling catheter and those who did not.

**Results:**

Of the 991 patients included, 498 (50.3%) underwent routine indwelling urinary catheter placement preoperatively. Routine indwelling catheterization was associated with a higher rate of urinary tract infection (1.4% vs 0.0%, *P* = .015), but a lower rate of minor POUR (5.0% vs 10.3%, *P* = .001). There was no difference with respect to the rate of major POUR or discharge with an indwelling catheter. Multivariate analyses demonstrated indwelling catheterization to be independently associated with a lower rate of minor POUR (*P* = .021), but there was no association with overall POUR, major POUR, or discharge with a urinary catheter.

**Conclusion:**

These data suggest that routine indwelling urinary catheterization is likely unnecessary for patients undergoing total hip arthroplasty in the setting of spinal anesthetic and may even lead to increased risk of complications such as urinary tract infection.

## Introduction

Postoperative urinary retention (POUR) represents a common complication following total hip arthroplasty (THA), with reported rates ranging from 6% to 84% [[1], [2], [3]]. While multiple factors often lead to the development of POUR, the use of spinal anesthesia has been theorized as a potential contributor secondary to neurologic blockade of the bladder, and it has been previously identified as an independent risk factor for retention in patients undergoing total joint arthroplasty [[4], [5], [6]]. Conversely, spinal anesthesia may have benefits with respect to an overall complication rate and length of stay when compared to general anesthesia for patients undergoing THA, which has led to a recent increase in the use of neuraxial blockade [[7], [8], [9]]. Urinary retention is not a benign complication following primary joint arthroplasty, however, as it has been associated with urinary tract infection (UTI), delayed mobilization, prolonged hospitalization, and increased readmission [1,10]. Furthermore, UTI has been has been identified as a source of bacteremia and associated risk factor for periprosthetic joint infection [2,11,12].

Controversy remains as to what constitutes best practice, with limited evidence to guide management. As such, some surgeons choose to implement prophylaxis against POUR in the form of routine indwelling urinary catheterization. Alternatively, other surgeons elect to avoid routine catherization in favor of close monitoring for development of retention, with intermittent catheterization used on an as-needed basis. While both routine indwelling catheterization and as-needed straight catherization represent viable options to prevent excessive bladder distension and long-term detrusor injury, each technique has had variable reported effects on the rate of POUR and UTI [2,3,10]. The purpose of this study was to examine the effect of routine indwelling urinary catheterization following primary THA performed under spinal anesthesia on the incidence of POUR and other urinary complications.

## Material and methods

Through examination of surgeon case logs, all patients who underwent primary THA under spinal anesthesia with one of two surgeons at a single academic medical center from 2014 through 2018 were identified. Complete medical records were obtained for all patients, and a retrospective chart review was performed. Data gathered included patient demographics, surgical time, volume of administered intraoperative fluids, anesthetic method, urinary catheterization, and postoperative urinary complications. Demographic data included age, sex, body mass index (BMI), tobacco use, ethnicity, history of benign prostatic hyperplasia, and burden of medical comorbidities as measured by the Charlson Comorbidity Index (CCI). Patients with chronic urinary retention or incontinence that required chronic indwelling catheterization or chronic self-catheterization were excluded.

All patients underwent primary THA via a direct anterior, direct lateral, or minimally invasive two-incision approach. The decision to utilize a perioperative indwelling urinary catheter was the decision of the operating surgeon, with one of the two included surgeons preferring regular use of catheterization and the other preferring no catheterization.

Patients who received an indwelling urinary catheter followed a standardized protocol, which involved removal of the catheter within 48 hours postoperatively. Catheter removal was typically performed the morning of postoperative day 1. All patients were monitored for the development of POUR based on patient-endorsed symptoms and through use of ultrasound bladder scans performed by nursing staff. This monitoring process began immediately postoperatively in patients who did not receive a urinary catheter and following catheter removal in patients who were catheterized. Patients who failed to void or had postvoid residuals with bladder scan readings greater than 400 milliliters were managed with straight catheterization. Up to 2 straight catheterizations were performed, after which point persistent retention was managed with indwelling catheter placement.

Patients who developed POUR were stratified into 2 tiers. Minor POUR was defined as retention that resolved following one or 2 straight catheterizations, while major POUR was defined as persistent retention following 2 straight catheterizations, which required postoperative indwelling catheter placement. All patients were monitored for dysuria and postoperative UTI, which was defined according to the National Surgical Quality Improvement Program diagnostic criteria within 30 days from surgery [12,13].

**Figure 1:**
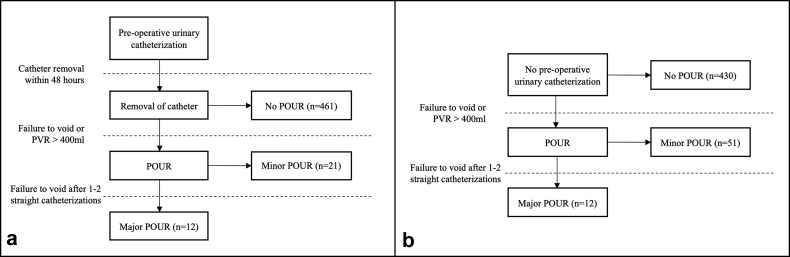
(a) Postoperative course for patients who received a routine indwelling urinary catheter. All catheters were removed within 48 hours postoperatively, with the standard being removal the morning of postoperative day 1. (b) Postoperative course for patients who did not receive a routine indwelling urinary catheter. In both cohorts, minor retention was defined as that which resolved following 1 or 2 straight catheterizations, while major retention was defined as that which persisted and required repeat indwelling catheter placement. POUR, postoperative urinary retention; PVR, postvoid residual.

Patients were divided into 2 groups: those who received a routine perioperative indwelling urinary catheter and those who did not. The postoperative protocol for each cohort is illustrated in Figure 1. Statistical analyses were performed to evaluate for differences between the 2 cohorts. Independent samples t tests were used for continuous variables, while chi-squared analyses and Fisher’s exact tests were used for categorical variables. Additionally, multivariate logistic regression analyses were performed to evaluate the effect of routine indwelling urinary catheterization on the rate of postoperative urinary retention after controlling for potential confounding variables. Covariates assessed in these multivariate analyses included age, sex, BMI, CCI, ethnicity, tobacco use, operative time, and intraoperative fluid volume. All statistical calculations were performed using IBM SPSS, version 26 (IBM Corporation, Armonk, NY), with significance set at *P* < .05 for all analyses.

## Results

A total of 1022 patients had primary THA under spinal anesthesia during the study period. Of these, 8 had a chronic indwelling urinary catheter or required chronic self-catheterization and were excluded. An additional 23 patients had incomplete data or inadequate postoperative follow-up and were excluded, as well. The remaining 991 patients comprised the final study group. Of these, 498 (50.3%) underwent routine indwelling urinary catheter placement preoperatively and comprised the “Catheter” cohort, while 493 (49.7%) comprised the “No Catheter” cohort. Use of an indwelling catheter was highly standardized by each surgeon, with 98.3% of patients in the “Catheter” group being treated by one surgeon and 98.2% of patients in the “No Catheter” group being treated by the other surgeon. Reasons for deviation from surgeon preference were irregularly recorded and not included in our analysis.

Full demographic and operative data are summarized in Table 1. There were no differences between the 2 groups with respect to age, BMI, CCI, history of benign prostatic hyperplasia, tobacco use, or ethnicity. The “No Catheter” cohort consisted of a greater proportion of female patients (57.4% vs 50.0%, *P* = .019). With respect to surgical data, patients in the “No Catheter” cohort had a longer operative time (118 vs 70 minutes, *P* < .0005) and received a greater volume of intraoperative fluids (1646 vs 1610 mL, *P* = .001).

**Table 1:** Demographic and operative data for patients who did and did not receive a routine indwelling urinary catheter.

Variable	No catheter (N = 493)	Catheter (N = 498)	*P*-value
Sex			.019^a^
Male	210 (42.6%)	249 (50.0%)	
Female	283 (57.4%)	249 (50.0%)	
Age (y)	61.5 ± 12.5	60.6 ± 13.9	.311
Body mass index	29.3 ± 5.6	29.4 ± 5.9	.783
Charlson Comorbidity Index	2.3 ± 1.6	2.2 ± 1.6	.351
Benign prostatic hyperplasia	12 (35.3%)	22 (64.7%)	.086
Tobacco use			.467
Smoker	41 (8.3%)	48 (9.6%)	
Nonsmoker	452 (91.7%)	450 (90.4%)	
Ethnicity			.341
Caucasian	416 (84.4%)	429 (86.1%)	
Black	32 (6.5%)	29 (5.8%)	
Hispanic	11 (2.2%)	13 (2.6%)	
Asian	22 (4.5%)	23 (4.6%)	
Other	12 (2.4%)	4 (0.8%)	
Surgical time (min)	118.2 ± 39.2	70.3 ± 26.0	<.0005^a^
Volume of intraoperative fluids (mL)	1646 ± 660	1610 ± 576	.001^a^

a Denotes variables that achieved statistical significance.

Patients who did not receive an indwelling urinary catheter had higher rates of overall POUR (12.8% vs 7.4%, *P* = .005) and minor POUR (10.3% vs 5.0%, *P* = .001). There was no difference between groups with respect to the rate of major POUR (2.4% vs 2.4%, *P* = .980) or discharge with an indwelling catheter (1.8% vs 1.8%, *P* = .983). Patients who received an indwelling urinary catheter had a higher rate of UTI (1.4% vs 0.0%, *P* = .015) and a longer length of hospital stay (2.2 vs 1.8 days, *P* < .0005). Postoperative urinary outcomes are summarized in Table 2.

**Table 2:** Outcomes data for patients who did and did not receive a routine indwelling urinary catheter.

Outcome	No catheter (N = 493)	Catheter (N = 498)	*P*-value
All POUR	63 (12.8%)	37 (7.4%)	.005^a^
Minor POUR	51 (10.3%)	25 (5.0%)	.001^a^
Major POUR	12 (2.4%)	12 (2.4%)	.980
Discharge with catheter	9 (1.8%)	9 (1.8%)	.983
Urinary tract infection	0 (0.0%)	7 (1.4%)	.015^a^
Length of stay (d)	1.8 ± 1.4	2.2 ± 1.4	<.0005^a^

POUR, postoperative urinary retention.

a Denotes variables that achieved statistical significance.

After controlling for potential confounding variables via multivariate analyses, use of an indwelling catheter was found to be independently associated with a reduced rate of minor POUR (odds ratio [OR] = 0.5, 95% confidence interval = 0.2-0.9, *P* = .021). Indwelling catheterization was found to have no association with the rate of overall POUR (*P* = .083), major POUR (*P* = .659), or discharge with an indwelling urinary catheter (*P* = .480). Given the low overall incidence of UTI, our data were inadequately powered to examine this variable via multivariate means. Data for all covariates that achieved significance in our multivariate analyses are provided in Table 3.

**Table 3:** Results of multivariate analyses examining the association between indwelling catheter use and postoperative retention.

	Exp(B)	95% confidence interval for Exp(B)	*P*-value
All POUR			
Indwelling catheterization	0.6	0.4-1.1	.083
Male gender	1.9	1.2-2.9	.004^a^
Minor POUR			
Indwelling catheterization	0.5	0.2-0.9	.021^a^
Male gender	1.8	1.1-3.0	.021^a^
Major POUR			
Indwelling catheterization	0.8	0.2-2.4	.659
Male gender	2.6	1.1-6.5	.038^a^
Increasing age	1.09	1.04-1.16	.001^a^

POUR, postoperative urinary retention.

Other covariates associated with each degree of retention are listed, as well.

a Denotes variables that achieved statistical significance.

## Discussion

Our data suggest that while routine indwelling urinary catheterization in patients undergoing THA who receive spinal anesthesia may have a protective effect against minor POUR, it has minimal effect on major retention and may possibly increase risk of UTI. Furthermore, indwelling catheterization was associated with an increased length of stay with no effect on the rate of discharge with a catheter.

Previous studies examining the effect of indwelling catheterization on outcomes following total joint arthroplasty have yielded conflicting results. A 2015 meta-analysis by Zhang et al. found indwelling catheterization to be associated with a lower rate of POUR with no effect on the rate of postoperative UTI in a cohort of patients undergoing both THA and total knee arthroplasty [14]. This is in contrast to Garbarino et al., who identified indwelling urinary catheterization as an independent risk factor for UTI in a retrospective review of over 7300 patients undergoing primary THA [15]. When considering primary THA performed under spinal anesthesia specifically, a randomized controlled trial of 200 patients by Miller et al. found no difference between indwelling as compared to intermittent catheterization with respect to POUR, UTI, or length of stay [3].

Our present study has several strengths over previous examinations of urinary complications following THA. First, to our knowledge, this represents the largest primary study group to date examining the effect of catheterization protocol on retention, with previous cohorts ranging from 200 to 719 patients [3,16]. Given the relatively low incidence of certain urinary complications such as UTI and discharge with a catheter, increased power is paramount in the identification of differences between groups. Next, few prior studies have examined urinary complications following THA under spinal anesthesia alone. Given the increasing prevalence of spinal anesthetic use in the total joint arthroplasty population, it is essential to identify any relevant effects of this technique [2,3,9]. This may be further emphasized by the increased incidence of same-day discharge THA, in which postoperative urinary complication or symptoms may be more difficult to safely evaluate. Lastly, our separation of retention into major and minor categories helps to better stratify the impact on each patient, as increased duration of catheterization represents an established risk factor for UTI [17].

Previously identified risk factors for POUR following total joint arthroplasty include use of spinal anesthesia, use of postoperative patient-controlled analgesia, increasing age, increasing intraoperative fluid administration, increasing time of indwelling catheterization, and history of urinary retention [5,6,10,18]. Lawrie et al. further identified higher volumes of intra-operative fluids and history of prior urinary retention as independent risk factors for POUR in patients undergoing THA who receive spinal anesthesia [2]. To complement these prior results, our study identified male gender as independently associated with major and minor retention and found increasing age to be independently associated with a higher risk of major retention. Furthermore, our results differed from those of prior studies in that we identified no association between intra-operative fluid levels and retention after controlling for possible confounders.

It has been theorized that indwelling catheterization may hinder patient recovery due to associated discomfort and hesitancy to mobilize with the catheter in place [6]. Such factors may have contributed to the increased length of stay observed in our “Catheter” cohort, as perhaps slower progress with physical therapy delayed discharge.

While it is a common complication following arthroplasty procedures, postoperative urinary retention itself remains poorly defined. Prior studies have used thresholds ranging from 300 to 800 milliliters on postoperative ultrasound bladder scans as diagnostic criteria for POUR and as an indication for catheterization [3,10,19]. This lack of consistency complicates interpretation of the literature. Regardless, a 2016 randomized controlled trial of 721 fast-track arthroplasty patients found a catheterization threshold of 800 milliliters to reduce the incidence of postoperative catheterization without increasing the rate of urological complications [19]. This suggests that current thresholds for diagnosis of POUR may be excessively sensitive and are thus capturing patients whose symptoms would otherwise resolve. Diagnostic criteria for POUR and thresholds for catheterization represent areas for future research.

While our results suggest as-needed straight catheterization to be a viable option, it is important to mention that success of intermittent catheterization as a treatment of POUR is dependent on close monitoring of patients by nursing staff in the postoperative period. Without close monitoring, unrecognized POUR can theoretically lead to severe bladder distension and permanent detrusor injury [4]. This has implications for smaller-volume settings where nursing staff may not be as familiar with monitoring for POUR. Furthermore, this has additional implications for same-day discharge arthroplasty programs, as patients must monitor for appropriate signs and symptoms on their own [6,20].

This study had several limitations. First, the retrospective nature of our study design and surgeon-specific adherence to an indwelling catheter or intermittent catheterization protocol introduced potential elements of bias. We attempted to control for these factors through use of multivariate analyses, but a randomized controlled trial would have superior methodology. Furthermore, the low incidence of UTI complicates its analysis with multivariate means, and future studies should strive to include a larger population of patients to improve power and eliminate this factor. Additionally, full anesthesia records were not available for all cases, preventing our study from controlling for medication and dosage used for spinal anesthesia.

## Conclusion

Our data suggest that routine indwelling urinary catheterization is likely unnecessary for patients undergoing THA in the setting of spinal anesthesia. Patients treated at centers with the capability to closely monitor for development of POUR are likely well served with as-needed straight catheterization given noninferior levels of major retention and possible reduced risk of UTI when compared to routine indwelling catheterization.

## Funding

This research did not receive any specific grant from funding agencies in the public, commercial, or not-for-profit sectors.

## Conflicts of interest

The authors declare the following financial interests/personal relationships which may be considered as potential competing interests: Paul A. Manner is the senior editor of the Clinical Orthopaedics and Related Research and is a representative of the AAOS, MEDCAC (Medicare Evidence Development & Coverage Advisory Committee), and Center for Medicare and Medicaid Services; all other authors declare no potential conflicts of interest.

For full disclosure statements refer to https://doi.org/10.1016/j.artd.2022.04.015.

## Statement of institutional review board approval

This study was approved by the University of Washington School of Medicine Institutional Review Board.

## Appendix A Supplementary data

Conflict of Interest Statement for Carlock

Conflict of Interest Statement for Fernando

Conflict of Interest Statement for Geiger

Conflict of Interest Statement for Manner

Conflict of Interest Statement for Mills
