# Supplementary material for: Routine Indwelling Urinary Catheterization Is Not Necessary During Total Hip Arthroplasty Performed Under Spinal Anesthesia
Source: Arthroplast Today. 2022 May 28;16:68–72. doi: 10.1016/j.artd.2022.04.015 (PMC9157006; doi:10.1016/j.artd.2022.04.015)
Supplement: Conflict of Interest Statement for Manner [file mmc4.pdf]

INDIVIDUAL CONFLICT OF INTEREST STATEMENT

American Association of Hip and Knee Surgeons

(Adopted from the American Academy of Orthopaedic Surgeons disclosure statement)

The following form **must be filled out completely and submitted by each author (example, 6 authors, 6 forms). All items require a response. If there is no relevant disclosure for a given item, enter "*None.*"**

Routine Indwelling Urinary Catheterization Is Not Necessary During Total Hip Arthroplasty Performed Under Spinal Anesthesia

Manuscript Title

1. Royalties from a company or supplier (The following conflicts were disclosed)

None

2. Speakers bureau/paid presentations for a company or supplier (The following conflicts were disclosed)

None

3A. Paid employee for a company or supplier (The following conflicts were disclosed)

None

3B. Paid consultant for a company or supplier (The following conflicts were disclosed)

None

3C. Unpaid consultants for a company or supplier (The following conflicts were disclosed)

None

4. Stock or stock options in a company or supplier (The following conflicts were disclosed)

None

5. Research support from a company or supplier as a Principal Investigator (The following conflicts were disclosed)

None

6. Other financial or material support from a company or supplier (The following conflicts were disclosed)

None

7. Royalties, financial or material support from publishers (The following conflicts were disclosed)

None

8. Medical/Orthopaedic publications editorial/governing board (The following conflicts were disclosed)

Senior Editor, *Clinical Orthopaedics and Related Research*

9. Board member/committee appointments for a society (The following conflicts were disclosed)
AAOS Representative, MEDCAC (Medicare Evidence Development & Coverage Advisory Committee), Center for Medicare and Medicaid Services

Each author must sign AND print or type his/her name, date and submit a separate form

In addition, one BLINDED Conflict of Interest form (no author names used) should be submitted per manuscript with all author disclosures.

Paul Manner, MD FRCSC [signature] 2/10/22

Author Name (Print or Type)	Author Signature	Date
